# Diagnostic and prognostic biomarkers in autoimmune hepatitis-associated cirrhosis: insights into TBil, CD38, IL-22, TSP-1, GAL-3, and Cyc-C

**DOI:** 10.3389/fmed.2025.1564107

**Published:** 2025-05-09

**Authors:** Jiani Shi, Jingbo Fu, Caijie He, Jun Shen

**Affiliations:** ^1^Daishan County First People's Hospital, Zhoushan, Zhejiang, China; ^2^Jiaxing Second Hospital, Jiaxing, Zhejiang, China

**Keywords:** autoimmune hepatitis (AIH), liver cirrhosis, cytokines, CD38, interleukin-22 (IL-22), biomarkers

## Abstract

**Background:**

Autoimmune hepatitis (AIH) is a chronic inflammatory disease that can progress to cirrhosis and liver failure. While various biomarkers have been associated with AIH progression and prognosis, their specific roles in diagnosing, predicting, and managing cirrhosis, particularly in compensated cirrhosis, remain unclear. Accurately predicting 2-year survival and clinical outcomes for patients with compensated cirrhosis is a critical challenge.

**Methods:**

This study analyzed clinical data from non-cirrhotic and cirrhotic AIH patients to identify factors influencing cirrhosis development and prognosis. Serum and nutritional indices were compared between groups with differing prognoses. The diagnostic efficacy of TSP-1, Gal-3, Cys-C, AIb, and PA was evaluated in compensated cirrhosis patients, and their prognostic value was validated using linear regression and Cox proportional hazards models.

**Results:**

No significant differences in gender, age, BMI, hypertension, diabetes history, or AMA positivity were observed between the non-cirrhotic AIH and cirrhotic AIH groups. However, TBil levels were significantly higher, and CD38 and IL-22 levels were significantly lower in the cirrhosis group. Multivariate logistic regression identified elevated TBil and reduced CD38 and IL-22 levels were associated with increased cirrhosis risk. Prognosis analysis revealed that patients with good outcomes had significantly lower TSP-1, Gal-3, and Cys-C levels and higher AIb and PA levels than those with poor outcomes. Cox proportional hazards modeling identified TSP-1, Gal-3, and Cys-C as independent risk factors and AIb and PA as protective factors influencing 2-year survival.

**Conclusion:**

This study underscores the critical roles of TBil, CD38, and IL-22 in cirrhosis development among AIH patients and validates the diagnostic and prognostic significance of TSP-1, Gal-3, Cys-C, AIb, and PA in compensated cirrhosis. These biomarkers facilitate early risk identification and inform clinical decision-making, contributing to improved management and outcomes for AIH patients.

## 1 Introduction

Autoimmune hepatitis (AIH) is a chronic liver disease characterized by immune-mediated hepatocellular damage, which can progress to cirrhosis if left untreated (1–3). Cirrhosis, the end-stage of liver fibrosis, is associated with significant morbidity and mortality, making its prevention and early detection critical for improving patient outcomes (4, 5). The pathogenesis of AIH involves a dysregulated immune response, where the liver is attacked by the body’s immune system (6). Although the disease affects individuals across all age groups, it is more commonly diagnosed in women, particularly those in their 50s and 60s.

The diagnosis of AIH is based on clinical, biochemical, immunological, and histological findings (7, 8). The 2022 American Academy of Hepatology Practice Guidelines provide a diagnostic framework for severe forms of liver disease, including decompensated cirrhosis, which can develop in AIH patients (9). These guidelines emphasize the use of imaging, biochemical, and physical assessments for accurate diagnosis. Management strategies for AIH primarily involve immunosuppressive therapy to control the immune response and prevent further liver damage (10). However, the progression to cirrhosis in AIH patients is highly variable, influenced by factors such as age, gender, body mass index (BMI), and comorbidities like hypertension and diabetes mellitus (11, 12). Antimitochondrial antibody (AMA) positivity is also assessed, as it may indicate alternative liver pathologies (13, 14). Despite these known factors, accurately predicting the development of cirrhosis in AIH patients remains a challenge.

In recent years, serum biomarkers have gained attention for their potential in predicting liver disease prognosis, including AIH (15, 16). Biomarkers such as Thrombospondin-1 (TSP-1), Galectin-3 (Gal-3), and Cystatin C (Cys-C) have been studied for their roles in assessing liver fibrosis and predicting cirrhosis progression (17–19). These biomarkers reflect various aspects of liver injury and function, providing valuable insights into disease severity and patient prognosis. This study aimed to investigate the clinical characteristics of AIH patients and identify factors influencing cirrhosis development.

## 2 Materials and methods

### 2.1 Research volunteer recruitment

The study adhered to the Declaration of Helsinki and received approval from the hospital’s Ethics Committee. And this study enrolled 75 patients with non-cirrhotic AIH and 18 patients with cirrhosis AIH hospitalized at our institution between June 2020 and June 2023. Clinical data, including gender, age, BMI, hypertension and diabetes history, AMA positivity, total bilirubin (TBil), aspartate aminotransferase (AST), alanine aminotransferase (ALT), white blood cell count (WBC), immunoglobulin G (IgG), CD38, and interleukin-22 (IL-22) levels, were analyzed to compare non-cirrhotic and cirrhotic groups. The non-cirrhotic cohort comprised 40 males and 35 females, aged 45–79 years, with a mean age of 59.31 ± 5.12 years. And the cirrhotic cohort comprised 10 males and 8 females, aged 42 to 80 years, with a mean age of 56.22 ± 6.28 years.

#### 2.1.1 Inclusion criteria

1. Met the diagnostic criteria for decompensated cirrhosis as outlined in the 2022 American Academy of Hepatology Practice Guidelines, confirmed through imaging, biochemical, and physical assessments. 2. No history of metabolic encephalopathy, toxic encephalopathy, or neurological disorders. 3. No active variceal bleeding within 1 month prior to admission. 4. Complete clinical data.

#### 2.1.2 Exclusion criteria

1. Patients with blood or infectious diseases. 2. Patients using immunosuppressants. 3. Patients with abnormal immune function. 4. Patients with other liver tumors. 5. Past recipients of liver transplantation. 6. Patients with severe heart disease.

### 2.2 Serum protein ELISA assay

A 5 mL fasting blood sample was collected from the cubital vein of each participant, and no repeated measures were taken. Serum was isolated by centrifugation at 2,000 rpm with a 6 cm radius for 3 min and stored at −80°C until analysis. Thrombospondin-1 (TSP-1), Galectin-3 (Gal-3), and Cystatin-C (Cys-C) levels were measured using enzyme-linked immunosorbent assay (ELISA) and a Hitachi Labospec T008AS automatic biochemical analyzer. Blood albumin (Alb) and prealbumin (PA) were analyzed using a Beckman Coulter LH750 total autokinetic blood cell analyzer.

### 2.3 Statistical analysis

Data analysis was performed using SPSS version 22.0. Variables following a normal distribution were expressed as mean ± standard deviation (SD) and analyzed using an independent *t*-test. The linear regression model was used to establish regression equations for analyzing correlations. The Cox proportional hazards regression model was employed to identify prognostic factors and develop a predictive model. The model’s accuracy was assessed using the area under the curve (AUC) of the receiver operating characteristic (ROC) curve. Survival rates were analyzed using Kaplan–Meier survival curves. A difference was considered statistically significant when *p* < 0.05.

## 3 Results

### 3.1 Comparison of clinical characteristics between patients in non-cirrhosis and cirrhosis group

To identify factors influencing cirrhosis development in patients with AIH, we analyzed clinical data from non-cirrhotic and cirrhotic patient groups (Table 1). No significant differences were observed between the two groups regarding gender, age, BMI, history of hypertension and diabetes, or AMA positivity. However, significant differences were found in total bilirubin (TBil) levels, which were notably higher in the cirrhotic group compared to the non-cirrhotic group. Levels of CD38 and IL-22 were significantly lower in the cirrhotic group. Multivariate logistic regression analysis identified TBil, CD38, and IL-22 as independent factors influencing cirrhosis development in AIH patients. Combined detection of TBil, CD38, and IL-22 levels demonstrated high sensitivity and specificity for predicting cirrhosis development in AIH patients, highlighting their potential as reliable biomarkers for early identification and risk assessment.

**Table 1:** Analysis of clinical data contrasting the good and poor prognosis groups.

Variable	Non-cirrhosis group (*n* = 75)	Cirrhosis group (*n* = 18)	Statistical value	*p*-value
Gender/cases (%)			χ^2^ = 0.029	0.865
Male	40(53.33)	10(55.56)		
Female	35(46.67)	8(44.44)		
Age/cases (%)			χ^2^ = 0.355	0.552
≤60 years	40(53.33)	11(61.11)		
>60 years	3(46.67)	7(38.89)		
BMI (kg/m^2^)	22.65 ± 2.04	22.61 ± 3.12	*t* = 0.067	0.947
Hypertension history/cases (%)			χ^2^ = 3.386	0.066
Present	11 (14.67)	6 (33.33)		
Absent	64 (85.33)	12 (66.67)		
Diabetes history/cases (%)			χ^2^ = 2.403	0.121
Present	16(21.33)	7(38.89)		
Absent	59(78.67)	11(61.11)		
AMA/cases (%)			χ^2^ = 0.000	1.000
Negative	0	0		
Positive	75(100.00%)	18(100.00%)		
TBil (μmol/L)	40.51 ± 16.34	85.91 ± 27.15	*t* = 9.183	0.001
AST (U/L)	53.26 ± 9.17	54.35 ± 10.29	*t* = 0.442	0.659
ALT (U/L)	59.39 ± 11.14	61.51 ± 12.28	*t* = 0.711	0.479
WBC (L)	5.51 ± 1.72 × 10^9^	5.87 ± 2.01 × 10^9^	*t* = 0.772	0.442
IgG (g/L)	19.46 ± 3.41	20.82 ± 3.75	*t* = 1.491	0.140
CD38 (ng/L)	9.24 ± 1.97	19.25 ± 3.04	*t* = 17.260	<0.001
IL-22 (ng/L)	71.28 ± 10.61	50.36 ± 7.09	*t* = 7.934	<0.001

BMI, body mass index; AMA, antimitochondrial antibody; TBil, total bilirubin; AST, aspartate aminotransferase; ALT, alanine aminotransferase; WBC, white blood cell; IgG, immunoglobulin G.

### 3.2 Comparative of serum and nutritional indexes between the good prognosis group and the poor prognosis group in non-cirrhosis patients

Serum and nutritional indicators were analyzed between the Good Prognosis and Poor Prognosis groups (Table 2). The Good Prognosis Group consisted of 38 subjects, while the Poor Prognosis Group had 37 subjects. The Good Prognosis Group exhibited significantly lower mean levels of Thrombospondin-1 (TSP-1), Galectin-3 (Gal-3), and Cystatin-C (Cys-C) compared to the Poor Prognosis Group. Additionally, the Good Prognosis Group had significantly higher mean levels of albumin (Alb) and prealbumin (PA). These findings demonstrate that the Good Prognosis Group exhibited notably superior serum and nutritional indicators relative to the Poor Prognosis Group.

**Table 2:** Comparison of serum and nutritional indicators between two groups.

Variable	Good prognosis (*n* = 38)	Poor prognosis (*n* = 37)	*t*-value	*P*-value
TSP-1(ng/mL)	1.11 ± 0.36	1.71 ± 0.33	7.288	<0.001
Gal-3 (ng/L)	17.77 ± 3.33	23.96 ± 3.91	7.719	<0.001
Cys-C (mg/L)	1.05 ± 0.23	1.54 ± 0.42	8.087	<0.001
AIb (g/L)	36.15 ± 4.48	28.89 ± 6.41	6.466	<0.001
PA (mg/L)	89.13 ± 10.42	72.53 ± 13.46	6.497	<0.001

TSP-1, thrombospondin-1; Gal-3, antimitochondrial antibody; Cys-C, cystatin C; AIb, albumin; PA, prealbumin.

### 3.3 Diagnostic performance analysis of biomarkers for predicting prognosis of patients with compensatory cirrhosis

We conducted an in-depth analysis to evaluate the diagnostic capabilities of biomarkers in compensated cirrhosis (Table 3). Our aim was to identify indicators that accurately forecast patient outcomes, thereby guiding clinical decisions and potentially enhancing patient outcomes. Our study assessed the diagnostic efficacy of key biomarkers. TSP-1 and Gal-3 emerged as strong predictors, with TSP-1 showing an area under the curve (AUC) of 0.892, sensitivity of 81.0%, and specificity of 100.0%. Gal-3 exhibited an AUC of 0.900, sensitivity of 90.5%, and specificity of 88.6%. These findings suggest that TSP-1 and Gal-3 have the potential to play a significant role in the early detection and prognosis of compensated cirrhosis. Furthermore, Cys-C, Alb, and PA also demonstrated promising diagnostic performance, with AUCs ranging from 0.832 to 0.885 and varying levels of sensitivity and specificity. Although these biomarkers are less robust than TSP-1 and Gal-3, they still offer valuable insights for clinical decision-making in compensated cirrhosis.

**Table 3:** Diagnostic performance of indicators in predicting outcomes of compensated cirrhosis.

Variable	Area	SE	*P*-value	95% Cl	Cut-off	Younden’s Index	Sensitivity	Specificity
TSP-1	0.892	0.055	<0.001	0.785–0.999	1.55	0.81	81.0	100.0
Gal-3	0.900	0.035	<0.001	0.831–0.969	20.95	0.791	90.5	88.6
Cys-C	0.885	0.051	<0.001	0.785–0.985	1.25	0.647	90.5	74.2
AIb	0.859	0.064	<0.001	0.733–0.984	31.4	0.804	85.7	94.7
PA	0.832	0.057	<0.001	0.720–0.943	82.1	0.63	85.7	77.3

95% Cl, 95% confidence interval; TSP-1, thrombospondin-1; Gal-3, antimitochondrial antibody; Cys-C, cystatin C; AIb, albumin; PA, prealbumin.

### 3.4 Relationship between linear regression analysis and prognosis of compensated cirrhosis

To investigate the relationship between patient prognosis in compensated cirrhosis and various biomarkers, we performed linear regression analysis (Table 4). The results showed that TSP-1, Gal-3, Cys-C, Alb, and PA were significantly correlated with prognosis in patients with compensated cirrhosis. TSP-1, Gal-3, and Cys-C were positively correlated with poor prognosis, while Alb and PA demonstrated a negative correlation with poor prognosis. Additionally, the tolerance and variance inflation factor (VIF) of each indicator are listed in the table to evaluate multicollinearity. These findings suggest that this biomarkers may be useful indicators for assessing the prognosis of patients with compensated cirrhosis.

**Table 4:** Relationship between linear regression analysis indicators and prognosis of compensated cirrhosis.

Indicator	Non-normalized coefficient		*β*	*t*	*P*-value	Tolerance VIF	
	R	Standard error				Tolerance	VIF
Constant	1.618	0.117	–	13.788	<0.001	–	–
TSP-1	0.126	0.046	0.149	2.749	0.007	0.543	0.543
Gal-3	0.033	0.004	0.386	7.633	<0.001	0.625	1.599
Cys-C	0.332	0.062	0.298	5.332	<0.001	0.513	1.950
AIb	−0.020	0.003	−0.315	−6.100	<0.001	0.599	1.669
PA	−0.010	0.001	−0.371	−7.266	<0.001	0.611	1.636

TSP-1, thrombospondin-1; Gal-3, antimitochondrial antibody; Cys-C, Cystatin C; AIb, albumin; PA, prealbumin.

### 3.5 Cox proportional hazards analysis of factors affecting 2-year survival rate in compensated cirrhosis

To investigate the impact of various biomarkers on the 2-year survival rate in patients with compensated cirrhosis, we conducted a Cox proportional hazards analysis (Table 5). TSP-1 significantly predicted survival, with a hazard ratio (HR) of 112.562 (95% CI: 31.739–399.203, *p* < 0.001), suggesting that elevated TSP-1 levels markedly increase mortality risk. Gal-3 (HR = 92.562, 95% CI: 12.392–691.394, *p* < 0.001) and Cys-C (HR = 43.773, 95% CI: 5.870–326.402, *p* < 0.001) were also significant risk factors for poor survival outcomes. Conversely, albumin (Alb) and prothrombin activity (PA) were associated with a reduced risk of mortality. The HR for Alb was 0.014 (95% CI: 0.003–0.059, *p* < 0.001), and for PA, it was 0.020 (95% CI: 0.003–0.147, *p* < 0.001), indicating that elevated levels of these biomarkers correlate with improved survival outcomes.

**Table 5:** Presents the Cox proportional hazards analysis identifying factors influencing the 2-year survival rate in patients with compensated cirrhosis.

Variable	β	S.E.	Wald χ^2^	*P*-value	OR (95% CI)
TSP-1	4.724	0.646	53.479	<0.001	112.562 (31.739–399.203)
Gal-3	4.528	1.026	19.477	<0.001	92.562 (12.392–691.394)
Cys-C	3.779	1.025	13.591	<0.001	43.773 (5.870–326.402)
AIb	−4.291	0.747	33.008	<0.001	0.014 (0.003–0.059)
PA	−4.291	0.747	33.008	<0.001	0.020 (0.003–0.147)

OR (95% CI), odds ratio (95% confidence interval); TSP-1, thrombospondin-1; Gal-3, antimitochondrial antibody; Cys-C, cystatin C; AIb, albumin; PA, prealbumin.

## 4 Discussion

Cirrhosis results from the prolonged destruction of the liver’s normal lobular structure due to various causes, leading to fibrosis, nodular regeneration, and hepatocyte necrosis (20–22). Decompensated cirrhosis represents an advanced stage of liver disease, characterized by portal hypertension and significant liver function impairment, with poor prognosis and high mortality (23). Liver transplantation remains the only curative option but is limited by high risk, cost, and insufficient donor availability. Thus, identifying effective and objective clinical indicators is crucial for treatment planning and prognosis evaluation (24).

Thrombospondin-1 (TSP-1) is a multifunctional trimeric protein involved in apoptosis, angiogenesis, and coagulation, playing a significant role in cirrhosis pathogenesis (25). Decreased liver function during decompensation alters TSP-1 production, making its serum levels a reliable marker for assessing disease severity and prognosis (26). This study identified elevated TSP-1 levels as strongly associated with poor prognosis, suggesting a positive correlation between high TSP-1 expression and adverse outcomes. Activation of TSP-1 precursor complexes can trigger signal transduction, causing extracellular matrix accumulation, reduced stromal degradation, and microenvironment changes, ultimately leading to fibrosis and microcirculatory dysfunction (27, 28). Additionally, TSP-1 acts as a potent activator of transforming growth factor-*β*1 (TGF-β1), binding to form the TSP1-LAP complex, which regulates cell proliferation and adhesion via signal transduction (29).

Galectin-3 is a β-galactoside-binding lectin extensively involved in inflammatory responses, apoptosis, and fibrotic processes (30–32). In cirrhosis, Galectin-3 is closely associated with the activation of hepatic stellate cells, a key step in liver fibrosis (33). Moreover, Galectin-3 affects the inflammatory response in the liver by regulating the infiltration of inflammatory cells and intercellular signaling (34). High expression of Galectin-3 may reflect the active degree of inflammation and fibrosis in cirrhotic patients, making it an important biomarker for assessing prognosis.

Cystatin-C (Cys-C) is a low-molecular-weight protein that serves as a marker of glomerular filtration rate due to its stable production and rapid metabolic breakdown (35, 36). This study found a positive correlation between elevated Cys-C levels and poor prognosis in decompensated cirrhosis. The proposed mechanism involves portal hypertension and splenomegaly, leading to immune and hematopoietic imbalances that elevate Cys-C levels. These findings align with prior studies linking Cys-C to disease severity. Malnutrition is a common complication in decompensated cirrhosis, driven by impaired liver function and gastrointestinal absorption (37).

Albumin (Alb) and prealbumin (PA) serve as key nutritional indicators, reflecting the liver’s synthetic capacity and the body’s nutritional status (38). This study found lower Alb and PA levels in patients with poor prognosis, indicating a negative correlation with survival outcomes. The mechanisms include increased energy consumption due to cirrhosis, reduced intake of high-quality proteins, and intestinal absorption disorders caused by portal hypertension, leading to malnutrition and decreased Alb and PA levels (39).

In the end, a Cox proportional hazards model identified independent risk factors influencing 2-year survival in decompensated cirrhosis:TSP-1 levels ≥1.55 ng/mL, Gal-3 levels ≥20.95 ng/L, Cys-C levels ≥1.25 mg/L, AIb levels <31.4 g/L, PA levels <82.1 mg/L. Our study demonstrated that TSP-1, Gal-3, and Cys-C independently increased the risk of death in compensated cirrhosis, whereas albumin and plasminogen activity could act as protective factors. These results highlight the potential clinical utility of these biomarkers in predicting survival and guiding therapeutic strategies in patients with compensated cirrhosis.

There are some limitations of our study, firstly, our study is a retrospective study, sample size is small and absence of external validation, future studies expand the sample size through multicenter collaboration to improve the statistical power and future external validation in independent cohorts to confirm the generalizability of our results. Secondly, our study was not externally validated, but we used appropriate models in our statistical analysis and plan to conduct external validation in future studies. Thirdly, our study focused on the assessment of short-term prognosis, the potential value of these biomarkers in long-term prognosis also warrants further exploration. Future studies will aim to validate the performance of these markers in long-term follow-up and explore their potential application in different disease stages.

## 5 Conclusion

In this study, TSP-1, Galectin-3, and Cystatin-C were found to be independent risk factors for death in patients with cirrhosis, whereas Albumin and Prealbumin were protective. The combined application of these markers provides a new tool for the assessment of short-term prognosis. However, this study has limitations, including a small sample size and the omission of other potential indicators such as vitamin B12, serum iron, and folic acid, which may introduce bias. Future research should address these limitations to enhance the reliability of findings and develop more comprehensive prognostic models.

## Data Availability

The original contributions presented in the study are included in the article/supplementary material, further inquiries can be directed to the corresponding author.

## References

[1] Kang Y Kuang X Yan H Ren P Yang X Liu H. A novel synbiotic alleviates autoimmune hepatitis by modulating the gut microbiota-liver Axis and inhibiting the hepatic TLR4/NF-κB/NLRP3 signaling pathway. mSystems. (2023) 8:e0112722. doi: 10.1128/msystems.01127-22, PMID: 36794950 PMC10134874

[2] Pellicano R Ferro A Cicerchia F Mattivi S Fagoonee S Durazzo M. Autoimmune hepatitis and fibrosis. J Clin Med. (2023) 12:1979. doi: 10.3390/jcm12051979, PMID: 36902767 PMC10004701

[3] Villani R Serviddio G Avolio C Cassano T D'Amico E. Autoimmune liver disease and multiple sclerosis: state of the art and future perspectives. Clin Exp Med. (2023) 23:3321–38. doi: 10.1007/s10238-023-01128-8, PMID: 37421590 PMC10618321

[4] Ginès P Krag A Abraldes JG Solà E Fabrellas N Kamath PS. Liver cirrhosis. Lancet. (2021) 398:1359–76. doi: 10.1016/S0140-6736(21)01374-X, PMID: 34543610

[5] Parola M Pinzani M. Liver fibrosis in NAFLD/NASH: from pathophysiology towards diagnostic and therapeutic strategies. Mol Asp Med. (2024) 95:101231. doi: 10.1016/j.mam.2023.101231 38056058

[6] Sipeki N Antal-Szalmas P Lakatos PL Papp M. Immune dysfunction in cirrhosis. World J Gastroenterol. (2017) 20:2564–77. doi: 10.3748/wjg.v20.i10.2564, PMID: 24627592 PMC3949265

[7] Sucher E Sucher R Gradistanac T Brandacher G Schneeberger S Berg T. Autoimmune hepatitis-immunologically triggered liver pathogenesis-diagnostic and therapeutic strategies. J Immunol Res. (2019) 2019:1–19. doi: 10.1155/2019/9437043 PMC6899271 31886312

[8] Chen H Han Z Fan Y Chen L Peng F Cheng X. CD4+ T-cell subsets in autoimmune hepatitis: a review. Hepatol Commun. (2023) 7:e0269. doi: 10.1097/HC9.0000000000000269, PMID: 37695088 PMC10497257

[9] Long MT Noureddin M Lim JK. AGA clinical practice update: diagnosis and Management of Nonalcoholic Fatty Liver Disease in lean individuals: expert review. Gastroenterology. (2022) 163:764–774.e1. doi: 10.1053/j.gastro.2022.06.023, PMID: 35842345 PMC9398982

[10] Strassburg CP. Autoimmune hepatitis. Dig Dis. (2013) 31:155–63. doi: 10.1159/000347211, PMID: 23797138

[11] Premkumar M Anand AC. Overview of complications in cirrhosis. J Clin Exp Hepatol. (2022) 12:1150–74. doi: 10.1016/j.jceh.2022.04.021, PMID: 35814522 PMC9257866

[12] Reau NS Lammert CS Weinberg EM. Autoimmune hepatitis: current and future therapies. Hepatol Commun. (2024) 8:e0458. doi: 10.1097/HC9.0000000000000458, PMID: 38836863 PMC11155538

[13] Lammert C Chalasani SN Green K Atkinson E McCauley B Lazaridis KN. Patients with autoimmune hepatitis report lower lifetime coffee consumption. Dig Dis Sci. (2022) 67:2594–9. doi: 10.1007/s10620-021-06989-1, PMID: 33939140 PMC8556390

[14] Klein R Hintz E Staehler G. Exacerbation of AIH in a patient with an AIH/systemic sclerosis overlap syndrome and pulmonary arterial hypertension treated with the endothelin-1 receptor antagonist sitaxentan. BMJ Case Rep. (2012) 2012:bcr0120125494. doi: 10.1136/bcr-01-2012-5494, PMID: 22802555 PMC4543042

[15] Tamber SS Bansal P Sharma S Singh RB Sharma R. Biomarkers of liver diseases. Mol Biol Rep. (2023) 50:7815–23. doi: 10.1007/s11033-023-08666-0, PMID: 37482588

[16] Di Sabatino A Biagi F Lenzi M Frulloni L Lenti MV Giuffrida P. Clinical usefulness of serum antibodies as biomarkers of gastrointestinal and liver diseases. Dig Liver Dis. (2017) 49:947–56. doi: 10.1016/j.dld.2017.06.010, PMID: 28733178

[17] Li Y Turpin CP Wang S. Role of thrombospondin 1 in liver diseases. Hepatol Res. (2017) 47:186–93. doi: 10.1111/hepr.12787, PMID: 27492250 PMC5292098

[18] Chen X Yu C Liu X Liu B Wu X Wu J. Intracellular galectin-3 is a lipopolysaccharide sensor that promotes glycolysis through mTORC1 activation. Nat Commun. (2022) 13:7578. doi: 10.1038/s41467-022-35334-x, PMID: 36481721 PMC9732310

[19] Assem NM Mohammed AI Barry HMA El Sayed IET Elmadbouh I. Serum cystatin C is an early renal dysfunction biomarker in patients with hepatitis C virus. Egypt Liver J. (2022) 12:67. doi: 10.1186/s43066-022-00231-x, PMID: 36466932 PMC9686297

[20] Dezső K Paku S Kóbori L Thorgeirsson SS Nagy P. What makes cirrhosis irreversible?-consideration on structural changes. Front Med. (2022) 9:876293. doi: 10.3389/fmed.2022.876293, PMID: 35572980 PMC9091510

[21] Tsochatzis EA Bosch J Burroughs AK. Liver cirrhosis. Lancet. (2014) 383:1749–61. doi: 10.1016/S0140-6736(14)60121-5, PMID: 24480518

[22] Çakıroğlu B. Minimally invasive connective water vapor energy method for benign prostatic hyperplasia. Urologia. (2024) 91:298–305. doi: 10.1177/03915603231216191, PMID: 38069654

[23] Tandon P Montano-Loza AJ Lai JC Dasarathy S Merli M. Sarcopenia and frailty in decompensated cirrhosis. J Hepatol. (2021) 75:S147–62. doi: 10.1016/j.jhep.2021.01.025, PMID: 34039486 PMC9125684

[24] Kappe NN Stolk J van Hoek B. Liver transplantation. N Engl J Med. (2024) 390:387. doi: 10.1056/NEJMc2314292, PMID: 38265663

[25] Bruel A Touhami-Carrier M Thomaidis A Legrand C. Thrombospondin-1 (TSP-1) and TSP-1-derived heparin-binding peptides induce promyelocytic leukemia cell differentiation and apoptosis. Anticancer Res. (2005) 25:757–64. PMID: 15868907

[26] Gwag T Reddy Mooli RG Li D Lee S Lee EY Wang S. Macrophage-derived thrombospondin 1 promotes obesity-associated non-alcoholic fatty liver disease. JHEP Rep. (2020) 3:100193. doi: 10.1016/j.jhepr.2020.100193, PMID: 33294831 PMC7689554

[27] Liu X Jin J Liu Y Shen Z Zhao R Ou L. Targeting TSP-1 decreased periodontitis by attenuating extracellular matrix degradation and alveolar bone destruction. Int Immunopharmacol. (2021) 96:107618. doi: 10.1016/j.intimp.2021.107618, PMID: 34015597

[28] Luo Q Jiang Z Jiang J Wan L Li Y Huang Y. Tsp-1+ microglia attenuate retinal neovascularization by maintaining the expression of Smad3 in endothelial cells through exosomes with decreased miR-27a-5p. Theranostics. (2023) 13:3689–706. doi: 10.7150/thno.84236, PMID: 37441592 PMC10334831

[29] Atanasova VS Russell RJ Webster TG Cao Q Agarwal P Lim YZ. Thrombospondin-1 is a major activator of TGF-β signaling in recessive dystrophic epidermolysis bullosa fibroblasts. J Invest Dermatol. (2019) 139:1497–1505.e5. doi: 10.1016/j.jid.2019.01.011 30684555

[30] Dong R Zhang M Hu Q Zheng S Soh A Zheng Y. Galectin-3 as a novel biomarker for disease diagnosis and a target for therapy (review). Int J Mol Med. (2018) 41:599–614. doi: 10.3892/ijmm.2017.3311, PMID: 29207027 PMC5752178

[31] Soares LC Al-Dalahmah O Hillis J Young CC Asbed I Sakaguchi M. Novel Galectin-3 roles in neurogenesis, inflammation and neurological diseases. Cells. (2021) 10:3047. doi: 10.3390/cells10113047, PMID: 34831271 PMC8618878

[32] Srejovic I Selakovic D Jovicic N Jakovljević V Lukic ML Rosic G. Galectin-3: roles in neurodevelopment, neuroinflammation, and behavior. Biomol Ther. (2020) 10:798. doi: 10.3390/biom10050798, PMID: 32455781 PMC7277476

[33] Pasmatzi E Papadionysiou C Monastirli A Badavanis G Tsambaos D. Galectin 3: an extraordinary multifunctional protein in dermatology. Current knowledge and perspectives. An Bras Dermatol. (2019) 94:348–54. doi: 10.1590/abd1806-4841.20198426, PMID: 31365668 PMC6668939

[34] Nangia-Makker P Hogan V Balan V Raz A. Chimeric galectin-3 and collagens: biomarkers and potential therapeutic targets in fibroproliferative diseases. J Biol Chem. (2022) 298:102622. doi: 10.1016/j.jbc.2022.102622, PMID: 36272642 PMC9706532

[35] Nishimura F Oniki K Miyamura S Ushijima T Harada H Taki T. Factors influencing glomerular filtration rate as estimated using preoperative creatinine and cystatin C levels. Int J Clin Pharmacol Ther. (2023) 61:363–70. doi: 10.5414/CP204432, PMID: 37347122

[36] Ermetici F Filopanti M Verga U Passeri E Dito G Malavazos AE. Estimated glomerular filtration rate by serum cystatin C correlates with cardiometabolic parameters in patients with primary hyperparathyroidism. Eur J Endocrinol. (2015) 173:441–6. doi: 10.1530/EJE-15-0341, PMID: 26194503

[37] Dent E Wright ORL Woo J Hoogendijk EO. Malnutrition in older adults. Lancet. (2023) 401:951–66. doi: 10.1016/S0140-6736(22)02612-5, PMID: 36716756

[38] Warner BW James JH Hasselgren PO Hummel RP Fischer JE. Effect of sepsis and starvation on amino acid uptake in skeletal muscle. J Surg Res. (1987) 42:377–82. doi: 10.1016/0022-4804(87)90172-7, PMID: 3553742

[39] Arroyo V Angeli P Moreau R Jalan R Clària J Trebicka J. Grifols chair and European Foundation for the Study of chronic liver failure (EF-Clif). The systemic inflammation hypothesis: towards a new paradigm of acute decompensation and multiorgan failure in cirrhosis. J Hepatol. (2021) 74:670–85. doi: 10.1016/j.jhep.2020.11.048, PMID: 33301825

